# Change in Physical Activity During the Coronavirus Disease 2019 Lockdown in Norway: The Buffering Effect of Resilience on Mental Health

**DOI:** 10.3389/fpsyg.2020.598481

**Published:** 2020-12-15

**Authors:** Frederick Anyan, Odin Hjemdal, Linda Ernstsen, Audun Havnen

**Affiliations:** ^1^Department of Psychology, Norwegian University of Science and Technology, Trondheim, Norway; ^2^Department of Public Health and Nursing, Norwegian University of Science and Technology, Trondheim, Norway

**Keywords:** COVID-19, confinement, physical activity, anxiety and depression symptoms, gender, age, resilience

## Abstract

Imposition of lockdown restrictions during the coronavirus disease 2019 (COVID-19) pandemic was sudden and unprecedented and dramatically changed the life of many people, as they were confined to their homes with reduced movement and access to fitness training facilities. Studies have reported significant associations between physical inactivity, sedentary behavior, and common mental health problems. This study investigated relations between participants’ reports of change in physical activity (PA; i.e., Reduced PA, Unchanged PA, or Increased PA) and levels of anxiety and depression symptoms during the COVID-19 pandemic lockdown in Norway in the time period from March 12, 2020 to June 15, 2020. The relations between age and gender and levels of anxiety and depression symptoms as well as how different levels of resilience influenced the relation between changes in PA and levels of anxiety and depression symptoms were also investigated. A cross-sectional survey design was used. Participants (*N* = 1,314; females = 31%) were members of an endurance sports organization aged between 18 and 81 years (*M* = 49 years; *SD* = 11.50 years). Participants completed the Resilience Scale for Adults and the Hospital Anxiety and Depression Scale and reported their changes in PA after lockdown restrictions were implemented on March 12, 2020. Regression analysis, independent samples *t*-test, and two-way multivariate analysis of variance were conducted. Reduced PA was associated with a higher risk of anxiety and depression symptoms. Younger participants in Reduced PA and Unchanged PA subgroups scored significantly higher on levels of anxiety symptoms and significantly higher on depression symptoms in Unchanged PA subgroup. Females in Unchanged PA and Increased PA subgroups scored significantly higher on levels of anxiety symptoms, whereas no gender differences were found for depression symptoms. The main and interaction effects of change in PA and resilience were significantly associated with depression symptoms. For anxiety symptoms, only the main effect of resilience, but not PA, and the interaction effect were significant. Results further showed that resilience was an important factor that influenced the levels of change in PA. High levels of resilience were associated with lower anxiety and depression symptoms in Reduced, Unchanged, and Increased PA subgroups during the COVID-19 lockdown. Promoting PA while boosting resilience factors such as confidence in own ability and drawing on the social support of even reduced social networks or connections while under lockdown can protect against common mental health problems.

## Introduction

The global outbreak of the novel coronavirus diseases [coronavirus disease 2019 (COVID-19)] caused by severe acute respiratory syndrome coronavirus 2 (SARS-COV-2) has become a major global health issue around the world. Following the declaration by the WHO that COVID-19 is a global pandemic, many countries including Norway imposed measures to mitigate the effects of COVID-19 and contain community spread of this highly infectious viral disease. The imposition of COVID-19 lockdown measures was sudden and unprecedented and dramatically changed the life of people, even globally, as it forced a global lockdown. Social connections and relationships were undermined due to restrictions on outdoor movements, social gatherings, and social distancing protocols. Many people were confined to their homes with reduced movement and access to fitness training facilities. Normal daily activities including physical activity (PA) were disrupted or significantly reduced. Stress, anxiety, and depression symptoms were common psychological reactions to the measures that were implemented to mitigate and contain the spread of COVID-19 (Rajkumar, 2020).

Several studies have investigated COVID-19-related adverse mental health outcomes (e.g., Brooks et al., 2020; Pfefferbaum and North, 2020; Rajkumar, 2020; Rossi et al., 2020). The consensus across these studies is that the global outbreak of the COVID-19 is a major stress that has negative consequences on people’s mental health (e.g., increases in post-traumatic stress symptoms, depression, anxiety, and insomnia). Rajkumar (2020) reported that evidence suggests anxiety and depression symptoms (16–28%) and self-reported stress (8%) are the commonest psychological reactions to the COVID-19 pandemic and may be associated with sleep problems. Additional evidence also suggests that the COVID-19 lockdown measures have caused significant changes in PA, which may explain the rise in mental health problems, including experiencing unpleasant emotions, sadness, anger, and frustrations (Lippi et al., 2020).

Regular participation in PA while under lockdown and confined to homes can be very challenging and almost impossible. Still, some amount of PA is recommended for preserving mental health. The WHO recommends that adults should engage in not less than 150 min per week of moderate-intensity aerobic PA, or not less than 75 min per week of vigorous-intensity aerobic PA, or an equivalent combination of moderate-and vigorous-intensity activity (WHO, 2020). Evidence from a meta-analysis of prospective cohort studies supports the preventive effect of PA on depression independent of age and geographic region (Schuch et al., 2018), and more recently, on anxiety, especially agoraphobia and post-traumatic disorder independent of demographic variables (Schuch et al., 2019). Previous studies have documented interesting results of PA. Among adults, PA has been found to be more effective for depression (Daley, 2008) and has potential benefits for reducing depression and anxiety among adolescents (Biddle and Asare, 2011). PA was found to be associated with a lower risk of mental health problems even in low doses (Teychenne et al., 2020), reducing depression by a medium effect [standardized mean difference (SMD) = −0.50; 95% CI: −0.93 to −0.06] and anxiety by a small effect (SMD = −0.38; 95% CI: −0.66 to −0.11) in nonclinical populations (Rebar et al., 2015). More recently, it has been found that while in lockdown due to COVID-19, light, moderate, and vigorous intensities of PA were associated with improved quality of life and decline in negative psychosocial effect of confinement (Slimani et al., 2020). Overall, mounting evidence suggests that not only does PA prevent and reduce anxiety and depression but also PA contributes to positive mental health and reduces the negative effects of COVID-19-related mental health problems (Schuch et al., 2018, 2019, 2020; Slimani et al., 2020; Teychenne et al., 2020).

Although it is well established that PA contributes to positive mental health (Rebar et al., 2015; Teychenne et al., 2020), the inability to participate in PA while under lockdown with restricted outdoor movements can negatively affect mental health. Despite significant changes to PA and the rise in COVID-19-related stress and mental health problems among some people due to the lockdown, other people with resilience factors or resources can overcome the odds for adaptive mental health (Wermelinger Ávila et al., 2017; Anyan, 2019). Resilience is the process and outcome of healthy adaptation despite significant stress (Fergus and Zimmerman, 2005). According to the resilience framework, despite the significant stress associated with COVID-19, not all people will show poor mental health (e.g., stress, depression, anxiety, and insomnia). On the contrary, some people will overcome the odds and preserve their mental health due to the availability of and access to resilience factors. Resilience has been found to be involved in the protection against anxiety and depression symptoms when exposed to major stress (Friborg et al., 2003; Anyan et al., 2017, 2019; Anyan, 2019) or when experiencing reduced or lack of social connections defined as loneliness (Anyan et al., 2020a). Protective factors involved in resilience represent intrapersonal and interpersonal resources that can modify the negative effects of stress and adversity on anxiety and depression symptoms (Anyan, 2019). Therefore, understanding how the COVID-19 pandemic has changed people’s PA and how resilience is involved in the protection against common mental health problems such as anxiety and depression symptoms seems warranted.

The COVID-19 pandemic also presents an important opportunity for research regarding the relations between relevant background characteristics (e.g., age and gender) and levels of symptom of anxiety and depression; hence, understanding the relations between age and gender and the levels of anxiety and depression symptoms seems warranted. The global prevalence of anxiety disorders across 44 countries identified age and gender among factors that accounted for the greatest variance, with younger adults and women more likely to have anxiety disorders (Baxter et al., 2013). Another review of the prevalence of depression in community samples across 30 countries identified sex among the factors that accounted for the greatest variance (Lim et al., 2018). Higher score of depression among women has been documented by other studies (Nolen-Hoeksema, 1990; Wolk and Weissman, 1995; Bebbington, 1996; Sprock and Yoder, 1997). Results from the relations between relevant background characteristics and common mental health problems during the COVID-19 lockdown can contribute to the body of knowledge on COVID-19 for intervention science and practice.

The first objective of this study was to investigate the relation between participants’ reports of change in PA (i.e., Reduced PA, Unchanged PA, or Increased PA) and anxiety and depression symptoms during the COVID-19 pandemic lockdown in Norway in the time period from March 12, 2020 to June 15, 2020. The second objective was to investigate the relations between age and gender and levels of anxiety and depression symptoms. Finally, this study investigated how different levels of resilience influenced the relation between change in PA and anxiety and depression symptoms.

Hypothesis i: It was expected that participants in the Reduced PA would be associated with a higher risk of anxiety and depression symptoms than participants in the Unchanged PA and Increased PA.
Hypothesis ii: It was expected that younger participants would score significantly higher on levels of anxiety and depression symptoms.
Hypothesis iii: Women were expected to score significantly higher on levels of anxiety and depression symptoms than men.
Hypothesis iv: It was expected that low resilience with reduced PA would be associated with higher levels of anxiety and depression symptoms.

## Materials and Methods

### Participants

This study was approved by The Regional Committees for Medical and Health Research Ethics board and the Norwegian Centre for Research Data. Participants provided a written informed consent. Invitation to participate in the study was sent to all (*N* = 6,766, males = 75%) members of an endurance sports organization by e-mail. The survey was administered via an online survey platform. A total of 1,317 persons answered the survey, and a final sample (*n* = 1,314; males = 69%) was included for analyses. Three participants who reported gender as “other” were removed from the data prior to analyzing hypothesized differences between women and men in the levels of anxiety and depression symptoms. Participants were aged between 18 and 81 years (*M* = 49 years; *SD* = 11.50 years). Additional background information of participants can be found in Table 1. Five participants did not report their gender, two did not report their educational level, and three did not report their marital status. Eight participants did not report their income.

**Table 1:** Background characteristics of all participants (*N* = 1,314).

Variable	Reduced, n (%) 183 (14%)	Unchanged, n (%) 845 (64%)	Increased, n (%) 286 (22%)
Background characteristics			
Age (years)	47	50	46
Gender, n (%)			
Females	65 (36%)	247 (29%)	93 (33%)
Males	118 (64%)	594 (71%)	192 (67%)
Marital status, n (%)			
Married	135 (74%)	694 (82%)	229 (80%)
Unmarried	48 (26%)	151 (18%)	57 (20%)
Education, n (%)			
Primary school	2 (1%)	7 (1%)	2 (1%)
Secondary school	28 (15%)	134 (16%)	33 (11%)
<4 years university	55 (30%)	233 (27%)	70 (25%)
≥4 years university	98 (54%)	469 (56%)	181 (63%)
Working, n (%)			
Yes	162 (88%)	731 (86%)	269 (94%)
No	21 (12%)	114 (14%)	17 (6%)
Income (×1,000)			
<250	1 (0.5%)	6 (1%)	0 (0%)
250–450	6 (3%)	27 (4%)	9 (3%)
451–750	36 (20%)	147 (17%)	46 (16%)
751–100	38 (21.5%)	146 (17%)	45 (16%)
>1,000	99 (55%)	514 (61%)	186 (65%)
History of mental disorder			
Yes	29 (16%)	83 (10%)	31 (11%)
No	153 (84%)	760 (90%)	255 (89)
	*M* (*SD*)	*M* (*SD*)	*M* (*SD*)
Resilience	4.78 (0.91)	5.03 (0.86)	5.11 (0.81)
HADS			
Anxiety	3.76 (3.47)	3.02 (2.93)	3.20 (2.88)
Depression	2.98 (2.93)	2.05 (2.29)	2.00 (2.06)

M, mean; SD, standard deviation; HADS, hospital anxiety and depression scale.

### Instruments

#### Background Characteristics

For recording background characteristics, participants were asked to provide information about age, gender, marital status, level of education, history of mental disorder, and if they were working.

#### Self-Reported Change in Physical Activity

One questionnaire item asked the participants to indicate how the imposition of lockdown restrictions due to the COVID-19 pandemic has impacted their PA routine. Participants selected from three nominal responses, namely, Reduced, Unchanged, and Increased PA.

#### Resilience

Two subscales from the Resilience Scale for Adults (RSA; Hjemdal et al., 2001; Friborg et al., 2003) were included for assessing levels of resilience. The RSA is a 33-item self-report questionnaire rated on a 7-point semantic differential scale format. Higher scores indicate higher levels of resilience protective factors. Cronbach’s alpha in this study was *α* = 0.83.

#### Symptoms of Anxiety and Depression

The Hospital Anxiety and Depression Scale (HADS; Zigmond and Snaith, 1983) includes 14 items rated on a 7-point Likert scale. The HADS was used to assess how participants felt over a 2-week period prior to measurement. HADS includes seven items that measure anxiety symptoms (HADS-A; *α* = 0.81) and seven items that measure depressive symptoms (HADS-D; *α* = 0.72). HADS performs well in assessing symptom severity in clinical settings and in the general population (Bjelland et al., 2002).

### Statistical Analyses

All analyses were conducted in IBM SPSS 25. Missing values for items were substituted with subscale mean scores. Similar approaches have been used elsewhere (Olstad et al., 2015; Anyan and Hjemdal, 2016, 2018). The first analyses computed the odds ratios (ORs) and 95% CI for the risk of anxiety or depressive symptoms per subgroup of PA using logistic regression (Hypothesis i). Simple linear regressions were conducted to investigate the effect of age on levels of anxiety and depression symptoms across all three different groups of change in PA (Hypothesis ii). Independent samples *t*-tests were used to investigate gender differences in the levels of anxiety and depression symptoms (Hypothesis iii). Finally, a two-way multivariate analysis of variance was used to investigate the main and interaction effects of resilience and change in PA on anxiety and depression symptoms (Hypothesis iv). Resilience scores were split at the median into low and high subgroups of resilience.

## Results

Table 1 displays background characteristics for Reduced PA, Unchanged PA, and Increased PA subgroups. Means and standard deviations of continuous measures and other relevant indicators are also displayed. The levels of anxiety and depression symptoms were generally low across all groups.

### Physical Activity Effect on Symptoms of Anxiety and Depression

Using HADS cutoff score (≥8), anxiety and depression symptoms were evident in 118 (9%) and 57 (4%) of the participants, respectively. Compared with the Increased PA, the Reduced PA was associated with a higher risk of anxiety (OR 2.77, 95% CI 1.510–5.086, *p* < 0.01) and depressive symptoms (OR 4.18, 95% CI 1.692–10.316, *p* < 0.01), but not the Unchanged PA after adjusting for age and gender.

### Age Effect on Symptoms of Anxiety and Depression Across Reduced, Unchanged, and Increased Physical Activity Subgroups

In the *Reduced PA* subgroup, regression of anxiety symptoms (standardized *β* = −0.23, *SE* = 0.02, *t* = −3.17, *p* < 0.01, *R*^2^ = 0.05) on age was significant, indicating that younger participants scored significantly higher on levels of anxiety symptoms. In the *Unchanged PA* subgroup, regression of anxiety symptoms (*β* = −0.20, *SE* = 0.01, *t* = −5.76, *p* < 0.001, *R*^2^ = 0.04) and depression symptoms (*β* = −0.10, *SE* = 0.01, *t* = −2.84, *p* < 0.01, *R*^2^ = 0.01) on age were all significant, indicating that younger participants scored significantly higher on levels of anxiety and depression symptoms. Regression of anxiety and depression symptoms on age in the *Increased PA* subgroup was not significant.

### Gender Differences in Symptoms of Anxiety and Depression Across Reduced, Unchanged, and Increased Physical Activity Groups

Females scored significantly higher than males on anxiety symptoms in both the *Unchanged PA* [(females: *M* = 3.58, *SD* = 3.45; males: *M* = 2.79, *SD* = 2.65), *t*(372.31) = 3.193, *p* < 0.01, mean difference (*MD*): 0.78 (95% CI: 1.264, 0.300)] and *Increased PA* [(females: *M* = 4.04, *SD* = 3.38; males: *M* = 2.81, *SD* = 2.52), *t*(143.19) = 3.115, *p* < 0.01, *MD*: 1.23 (95% CI: 2.011, 0.449)] subgroups. No significant gender differences in depression symptoms were found in all the groups and in anxiety symptoms for the *Reduced PA* subgroup.

### Differences in Change in Physical Activity and Resilience on Symptoms of Anxiety and Depression

**Figure 1:**
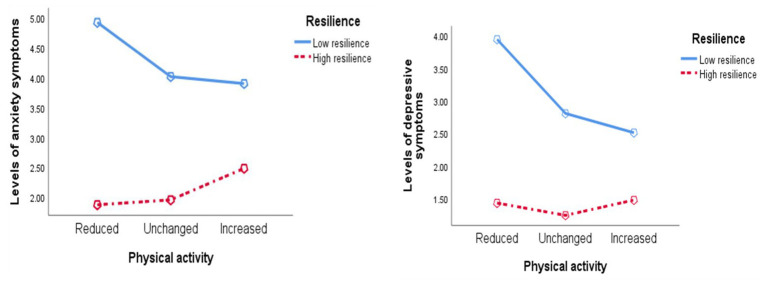
The relations between physical activity and resilience on anxiety and depression symptoms.

When predicting the levels of anxiety symptoms, significant main effects were found for resilience, *F*(1, 1,307) = 129.32, *p* < 0.001, partial *η*^2^ = 0.09, but not self-reported change in PA, *F*(2, 1,307) = 1.81, *p* = 0.165. However, the interaction effect was significant *F*(2, 1,307) = 4.56, *p* < 0.05, partial *η*^2^ = 0.01. Follow-up univariate tests indicated significant effects of resilience within each level combination of self-reported change in PA (all *p* < 0.001). Specifically, the levels of anxiety symptoms were higher when scoring low on resilience in the *Reduced PA* subgroup (*MD* = 3.06; 95% CI: 2.218, 3.897), *Unchanged PA* (*MD* = 2.07; 95% CI: 1.687, 2.447), and *Increased PA* (*MD* = 1.42; 95% CI: 0.768, 2.074). When predicting the levels of depression symptoms, significant main effects were found for resilience, *F*(1, 1,307) = 128.983, *p* < 0.001, partial *η*^2^ = 0.09, and self-reported change in PA, *F*(2, 1,307) = 6.99, *p* < 0.01. The interaction effect was significant, *F*(2, 1,307) = 6.11, *p* < 0.01, partial *η*^2^ = 0.01. Follow-up univariate tests indicated significant effects of resilience within each level combination of self-reported change in PA (all *p* < 0.001). Specifically, the levels of depression symptoms were higher when scoring low on resilience in the *Reduced PA* subgroup (MD = 2.52; 95% CI: 1.861, 3.176), *Unchanged PA* (MD = 1.57; 95% CI: 1.270, 1.865), and *Increased PA* (MD = 1.04; 95% CI: 0.524, 1.546; Figure 1).

## Discussion

This study investigated relations between participants’ reports of change in PA (i.e., Reduced PA, Unchanged PA, or Increased PA) and anxiety and depression symptoms during the COVID-19 pandemic lockdown in Norway. The relations between age and gender and levels of anxiety and depression symptoms as well as how different levels of resilience influenced the relation between change in PA and anxiety and depression symptoms were also investigated. Results from the descriptive statistics showed that participants in the Reduced PA subgroup reported the highest levels of anxiety and depression symptoms and the lowest on resilience. Conversely, participants in the Increased PA subgroup reported the lowest levels of depression symptoms and the highest on resilience. In support of the first hypothesis, Reduced PA (but not Unchanged PA) was associated with higher odds of caseness anxiety and depression symptoms than Increased PA. This finding is not surprising, as recent evidence continues to show that PA is associated with a lower risk of mental health problems (Teychenne et al., 2020) and reduction in levels of anxiety and depression (Daley, 2008; Biddle and Asare, 2011). In the wake of the COVID-19 lockdown, other studies have reported that the lack of PA during COVID-19 lockdown could be associated with experiencing unpleasant emotions, sadness, anger, and frustrations (Lippi et al., 2020), which may explain why the Reduced PA was associated with a higher risk of caseness anxiety and depression symptoms. It is, therefore, interesting that the present findings show that, despite the lockdown, participants who were in the Increased PA subgroup reported the lowest scores on anxiety and depression symptoms and the highest score on resilience. Thus, PA could contribute to preserve mental health and reduce the negative effects of anxiety and depression while boosting levels of resilience during the lockdown.

The second hypothesis sought to determine the relation between age and the levels of anxiety and depression symptoms. It was expected that younger participants would report significantly higher levels of anxiety and depression symptoms. Different results were found between the three groups of change in PA. Specifically, in the Unchanged PA subgroup, younger participants scored significantly higher on both anxiety and depression symptoms, while in the Reduced PA subgroup, younger participants scored significantly higher on only anxiety symptoms. This finding partly corresponds to results from an Australian study that found higher scores in one or more psychological distress states among the youngest age group (18–45 years) during the COVID-19 lockdown (Stanton et al., 2020). There is little consensus in the literature describing what happens to the risk of anxiety and depression as people get older, and the empirical evidence is largely variable. Older reviews of epidemiological data indicated that there was no consistent pattern across studies for the age differences in anxiety and depression (Jorm, 2000). Furthermore, Jorm (2000) reported that age biases in the assessment of anxiety and depression and the masking effect of other risk factors account for the observed common trend whereby an initial rise in the symptoms across age was followed by a drop in symptoms (Jorm, 2000).

The main conclusion in a later meta-analytic review was that older adults were about 20% less likely to be diagnosed with anxiety compared with younger adults (Baxter et al., 2013), suggesting that old age reduces the risk of anxiety and depression. Supporting Baxter’s conclusion and consistent with findings in this study, evidence from previous empirical studies have found significant negative associations between age and anxiety and depression symptoms among a sample of adults in the general population (Anyan et al., 2017). However, other studies found no associations among a university sample (Anyan et al., 2020b), adults in a community dwelling (Strawbridge et al., 2002), and adults undergoing chemotherapy (Weiss Wiesel et al., 2015). Overall, our cautious interpretation of the findings is that higher age may reduce the risk of anxiety and depression independent of change in PA, although the stability of this finding across different developmental stages cannot be defended. As such, this finding should be further investigated across different developmental periods in a life span perspective using longitudinal studies.

Hypothesis iii sought to determine the relations between gender and the levels of anxiety and depression symptoms. Women were expected to report higher levels of anxiety and depression than men. Meta-analytic reviews have found that women report significantly higher than men on anxiety (Baxter et al., 2013) and depressive symptoms (Nolen-Hoeksema, 1990; Wolk and Weissman, 1995; Bebbington, 1996; Sprock and Yoder, 1997; Lim et al., 2018), which was the basis for our second hypothesis. Various explanations have sought to clarify the consistently high scores of anxiety and depression among women, attributable to risk factors including genetic, hormonal, psychological, and psychosocial variables (Kuehner, 2003). A recent study also found women to have significantly higher scores than men in one or more psychological distress states during the COVID-19 lockdown (Stanton et al., 2020). The findings in this study corroborate the differences in levels of anxiety, but not depressive symptoms. Women scored significantly higher on anxiety symptoms in the Unchanged PA and Increased PA groups, but not in the Reduced PA group. Contrary to our hypothesis, women did not have elevated depression scores compared to men in any of the PA groups. However, in some aspects, particularly for the Reduced PA, the results are in line with a large Norwegian population study that did not find gender differences for the association between cardiorespiratory fitness, a physiological measure highly influenced by PA level, and depression and anxiety (Shigdel et al., 2019). The same study indicated a positive effect of cardiorespiratory fitness on depression, but not for anxiety. This finding is supported by a meta-analysis that showed that PA has a stronger antidepressive effect than for anxiety (Rebar et al., 2015). Possible explanations for the findings in the present study could be that depressed women did not participate in the online survey (only 31% of the sample were women, whereas 69% were men), and that highly physically active women reduced the risk for depression, but levels of anxiety were less influenced by PA. Another important characteristic of the present study is the generally high sociodemographic status of the participants. The majority of respondents had a high level of education, high income, and were married, all of which may protect against mental illness. It must be noted though that other studies show an association between PA and anxiety (Brunes et al., 2015; Schuch et al., 2019), and the failure to establish such a relationship in the present study warrants further research.

The fourth hypothesis sought to determine how different levels of resilience influenced the relation between change in PA and anxiety and depression symptoms. Restrictions during the COVID-19 pandemic caused significant stress with mental health problems (Brooks et al., 2020; Pfefferbaum and North, 2020; Rajkumar, 2020; Rossi et al., 2020), and as such, resilience could be an important factor that may contribute to help people preserve mental health despite the stress accompanying COVID-19 and the lockdown measures. At the general level, findings in this study showed that the levels of anxiety and depressive symptoms were higher when scoring low on resilience in the Reduced PA than in the Unchanged PA and Increased PA, respectively. Alternatively, the levels of both anxiety and depressive symptoms were higher among low resilience participants who reduced their PA due to COVID-19 lockdown than among participants who increased their PA. This result brings into relief the benefits of PA and resilience during a pandemic. Recent evidence shows that while in lockdown due to COVID-19, participation in PA was associated with improved quality of life and decline in negative psychosocial effect due to confinement (Slimani et al., 2020).

Resilience-based interventions have delivered promising results for adaptive mental health, thus becoming an auspicious initiative for mental health intervention and practice (Anyan et al., 2018; Anyan, 2019). When facing pandemics with major restrictions and lockdowns, people could benefit from participation in PA and could even benefit more in combination with improving access to protective resources involved in resilience. For example, promoting PA while boosting resilience factors, such as confidence in own ability and drawing on the social support of even the reduced social networks or connections while under lockdown, may protect against stress and common mental health problems due to the outbreak of a sickness such as COVID-19. The findings in this study deepen the body of knowledge related to the consequences of PA and adaptive psychosocial and behavioral factors that can contribute to various intervention responses to COVID-19. Overall, findings in this study also contribute to the growing body of primary research and identifying the nature of relations between PA, resilience factors, and common mental health problems, especially during pandemics.

The findings should be interpreted in the light of some limitations. The three groups of change in PA were highly disproportional in their sample sizes, with only 14% of the total sample belonging to the Reduced PA subgroup while 64% belonged to the Unchanged PA and 22% in the Increased PA, which may relatively affect the power to detect effects in the analyses. The use of self-report survey questionnaires without further clinical observations only indicates the levels of symptoms without implications for clinical diagnosis. As such, generalizing results to clinical samples may be problematic. As the study sample consisted of members from a fitness association, it is not surprising that they reported a generally high weekly PA level. In addition, the fact that the average education level was very high, the findings may not be representative of the general population. The fitness association had a majority of male members (69% in total), which is reflected in the overrepresentation of male respondents in the survey, and this limits the generalizability of the results to women. Self-report survey questionnaires are also vulnerable to social desirability, which could be a potential problem for a study that relied on self-reported retrospective behaviors and thoughts. The use of cross-sectional samples precludes answering questions about protective mechanisms or processes involved in resilience, as longitudinal studies are better suited for answering questions about processes and mechanisms (Anyan and Hjemdal, 2016).

## Data Availability Statement

The datasets presented in this article are not readily available because the ethics requirement is to keep the data under lock and key. Requests to access the datasets should be directed to frederick.anyan@ntnu.no.

## Ethics Statement

The studies involving human participants were reviewed and approved by The Regional Committees for Medical and Health Research Ethics board and the Norwegian Centre for Research Data. The patients/participants provided their written informed consent to participate in this study.

## Author Contributions

FA contributed to the conceptualization of study, statistical analyses and interpretation of results and drafted and made substantive edits and revisions to the manuscript. OH provided feedback and contributed to revising the manuscript through either direct edits or feedback. LE and AH contributed to the conceptualization of study design and made substantive edits and revisions to the manuscript. All authors contributed to the article and approved the submitted version.

### Conflict of Interest

The authors declare that the research was conducted in the absence of any commercial or financial relationships that could be construed as a potential conflict of interest.
